# Expression and role of VLA-1 in resident memory CD8 T cell responses to respiratory mucosal viral-vectored immunization against tuberculosis

**DOI:** 10.1038/s41598-017-09909-4

**Published:** 2017-08-25

**Authors:** Siamak Haddadi, Niroshan Thanthrige-Don, Sam Afkhami, Amandeep Khera, Mangalakumari Jeyanathan, Zhou Xing

**Affiliations:** 1 McMaster Immunology Research Centre, Department of Pathology & Molecular Medicine, Hamilton, Ontario Canada; 2 Michael G. DeGroote Institute for Infectious Disease Research, McMaster University, Hamilton, Ontario, Canada

## Abstract

Lung resident memory T cells (T_RM_) characterized by selective expression of mucosal integrins VLA-1 (α1β1) and CD103 (α_E_β7) are generated following primary respiratory viral infections. Despite recent progress, the generation of lung T_RM_ and the role of mucosal integrins following viral vector respiratory mucosal immunization still remains poorly understood. Here by using a replication-defective viral vector tuberculosis vaccine, we show that lung Ag-specific CD8 T cells express both VLA-1 and CD103 following respiratory mucosal immunization. However, VLA-1 and CD103 are acquired in differential tissue sites with the former acquired during T cell priming in the draining lymph nodes and the latter acquired after T cells entered the lung. Once in the lung, Ag-specific CD8 T cells continue to express VLA-1 at high levels through the effector/expansion, contraction, and memory phases of T cell responses. Using a functional VLA-1 blocking mAb, we show that VLA-1 is not required for trafficking of these cells to the lung, but it negatively regulates them in the contraction phase. Furthermore, VLA-1 plays a negligible role in the maintenance of these cells in the lung. Our study provides new information on vaccine-inducible lung T_RM_ and shall help develop effective viral vector respiratory mucosal tuberculosis vaccination strategies.

## Introduction

Immunological memory acquired following natural infection or immunization has a critical role in host defence against infectious diseases. T cell immune responses induced by natural infection or immunization persists in the form of effector (T_EM_) or central (T_CM_) memory T cells^1^. In the recent years it has become clear that some of the effector memory T cells reside in non-lymphoid tissues, the site of infection, following pathogen clearance and are considered as non-circulating memory cells named resident memory T cells (T_RM_) which play a critical role in immune protection^2–6^.

T_RM_ are typically defined by the expression of surface markers including integrin molecules. Interaction of integrins on T cells with extracellular matrix proteins is believed to play a critical role in T cell trafficking and retention in non-lymphoid mucosal tissues^7, 8^. Furthermore, integrin molecules have also been implicated in regulation of T cell differentiation^9, 10^ and survival-related signalling pathways^11^. In this regard T_RM_ persisting in the lung after acute respiratory viral infection selectively express integrins α_1_β_1_ (also known as VLA-1/CD49a) and α_E_β_7_ (CD103), as well as early-activation marker CD69, and provide robust protection against subsequent infections^5, 6^. In particular, abundant VLA-1-expressing T_RM_ were induced in murine lungs by influenza infection, and VLA-1 was shown to play a role in retention and survival, but not in trafficking, of influenza-specific CD8 T cells in the lung^12, 13^. The VLA-1-expressing T_RM_ have also been seen in human lungs and such lung T_RM_ appear unique in that they differ from their skin and gut counterparts in their frequency^6, 14, 15^. Nevertheless, much still remains to be understood about the development of T_RM_ and the functional role of T_RM_-associated integrins such as VLA-1 in the lung following respiratory mucosal viral infection.

Viral vector respiratory mucosal route of immunization has emerged as a new strategy for generating effective protective immunity against mucosal pathogens such as *Mycobacterium tuberculosis* ^16–18^ and enhanced knowledge in vaccine-induced lung T_RM_ will help improve such strategies. Among the most promising respiratory viral vector vaccines are the recombinant human or chimpanzee adenovirus, MVA and sendai virus expressing selected immunodominant microbial antigens shown to be protective against mucosal infections including tuberculosis (TB), RSV, HIV or herpes virus^19–25^. These viral vectors are designed to be replication-defective for improved safety and are yet capable of infection to induce long-lasting T cell responses^26^. However, many differences exist in the immune responses elicited to various viral species, and replication effective (wild type) vs replication-deficient viral infections, and differential innate immune activation, and antigenic expression can all influence T_RM_ generation^27^. Up to date, it still remains to be determined whether replication-defective viral vector respiratory mucosal immunization induces T_RM_ in the lung and what are the functional roles of integrin molecules in the regulation of T_RM._

In the current study we have used a replication-defective adenovirus-vectored TB vaccine to investigate the T_RM_ properties of respiratory mucosal immunization-induced Ag-specific T cells and the role of T_RM_-associated integrin VLA-1 in such T cell responses. Our study shows that replication-defective viral vector respiratory mucosal TB immunization promotes lung T_RM_ generation. However, T_RM_ integrins, VLA-1 and CD103, were acquired in different phases of T cell responses and differential tissue sites. We further show that VLA-1 was not involved in T cell trafficking to the lung but rather, it played a regulatory role in the contraction phase of T cell responses in the lung. Furthermore, we found that VLA-1 is dispensable for T_RM_ maintenance during the memory phase.

## Results

### Viral-vectored respiratory mucosal immunization induces Ag-specific CD8 T cells in the lung with distinct gene expression profile

**Figure 1:**
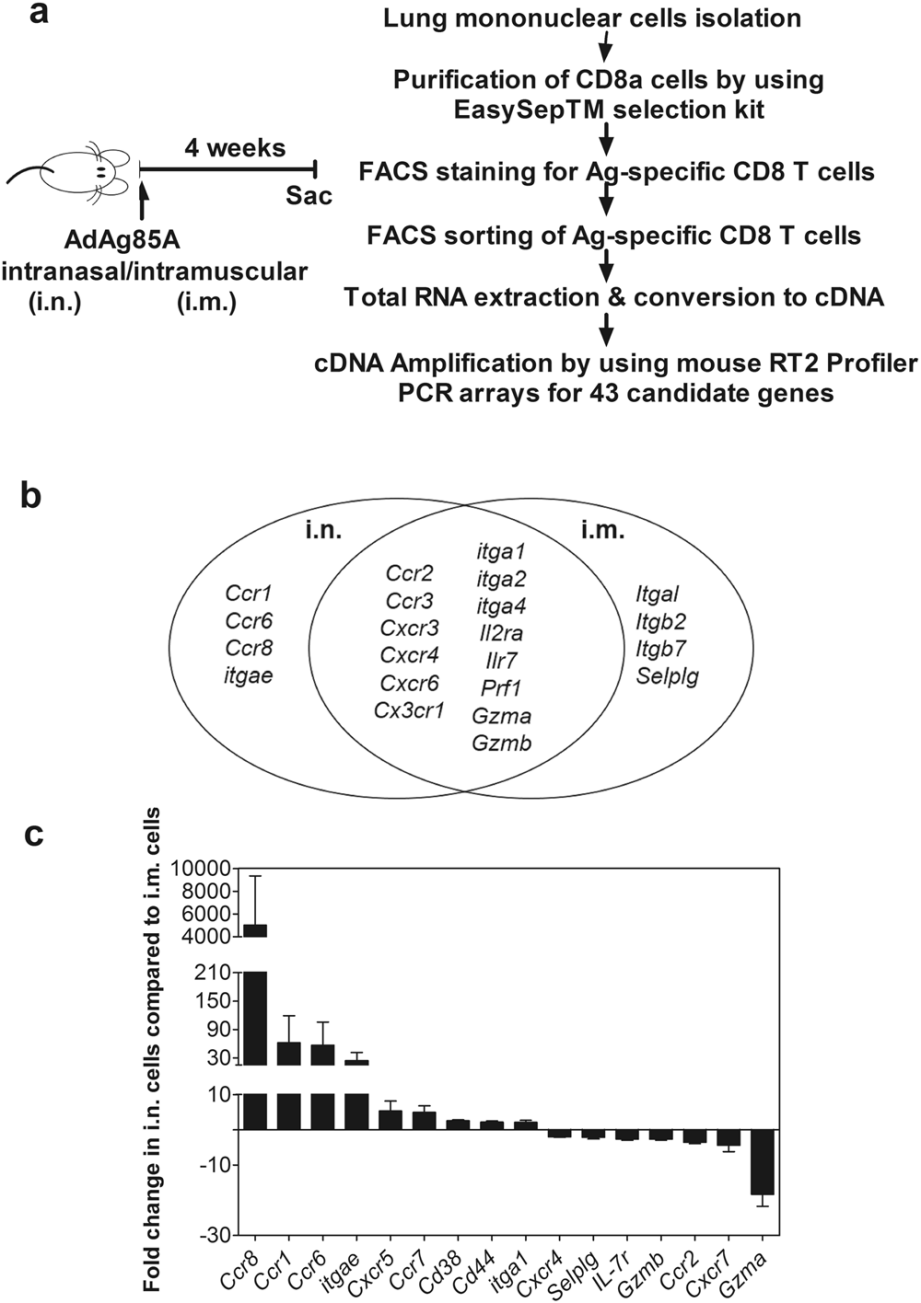
Expression of candidate genes by Ag-specific CD8 T cells induced by replication-defective viral-vectored respiratory mucosal immunization. (**a**) Experimental schema and flow chart showing the workflow. (**b**) Venn diagram depicts genes that are commonly expressed on both respiratory mucosal (i.n.) and parenteral intramuscular (i.m.) immunization-induced Ag-specific CD8 T cells, and the genes that are uniquely expressed on i.n.- and i.m.-immunization induced Ag-specific CD8 T cells. (**c**) Bar graph shows mean ± S.E.M. fold changes of genes expressed by i.n. immunization-induced Ag-specific CD8 T cells compared to i.m. immunization-induced Ag-specific CD8 T cells. Data represent mean fold changes calculated from 3 independent experiments.

To begin investigating whether respiratory mucosal immunization promotes lung T_RM_ generation, we first set out to characterize the properties of vaccine-induced Ag-specific CD8 T cells in the lung. An adenovirus-vectored tuberculosis vaccine (AdAg85A) was used as a model replication-defective viral vector vaccine and this vaccine, when delivered via respiratory mucosal and parenteral intramuscular routes, induced Ag-specific CD8 T cell responses in the lung^21, 28^. Interestingly, using intravascular immunostaining it has recently been shown that >95% of T cells in a naive lung are trapped in the pulmonary vasculature and bona fide lung tissue T cells were detected only after respiratory mucosal infection or immunization^29, 30^. Thus using such intravascular immunostaining we first verified Ag-specific T cell distribution in the lung at 4 weeks following respiratory mucosal and parenteral route of immunization. We found that the vast majority of Ag-specific CD8 T cells induced by respiratory mucosal immunization were bona fide lung tissue T cells. In sharp contrast, most of the Ag-specific CD8 T cells induced by parenteral intramuscular immunization were located in the lung vasculature. To determine the unique properties of respiratory mucosal immunization-induced (i.n.) lung tissue Ag-specific memory CD8 T cells, we compared gene expression of these cells with gene profile in parenteral AdAg85A immunization-induced (i.m.) intravascular Ag-specific CD8 T cells at 4 weeks post-immunization and in naïve CD8 T cells. Such comparisons help identify the genes commonly induced by both routes of immunization and those uniquely expressed in respiratory mucosal immunization-induced lung Ag-specific CD8 T cells. Genes encoding for chemokine receptors, integrin heterodimers, and some activation makers (Supplementary Table 1) implicated in T cell trafficking, maintenance and differentiation^8^ were profiled in FACS sorting-purified Ag-specific (Ag85A-tetramer-positive) CD8 T cells (CD8+tet+T cells) by using a custom-made PCR array (Fig. 1a). The relative gene expression was determined using data from the real-time cycler and the ΔΔCT method as previously described^31^. For our first goal, that is, identifying gene profile related to immunization, we focused on comparison of i.n. with control and i.m. with control (Fig. 1b). We found a set of genes commonly induced in Ag-specific CD8 T cells by both i.n. and i.m. immunization compared to naïve CD8 T cells (Fig. 1b). However, the genes encoding proteins CCR1, CCR6, CCR8, and CD103 (*itage*) were uniquely induced in i.n. immunization-induced CD8 T cells (Fig. 1b). Levels of *Ccr1, Ccr6, Ccr8* and *itage* gene expression by i.n. immunization-induced T cells were at least 30-fold higher than those by i.m. immunization (Fig. 1c). In addition, expression of *Cxcr5, Ccr7, CD34*, *CD44* and *itga1* (α1 integrin of VLA-1 or CD49a) genes also increased by more than 2 fold in i.n. immunization-induced memory CD8 T cells (Fig. 1c). Taken together, these data indicate that viral vector mediated respiratory mucosal TB immunization induces lung tissue Ag-specific memory CD8 T cells with a unique set of genes that are implicated in T cell mucosal tissue trafficking and maintenance.

### Viral-vectored respiratory mucosal immunization induces Ag-specific CD8 T cells expressing T_RM_ surface markers

**Figure 2:**
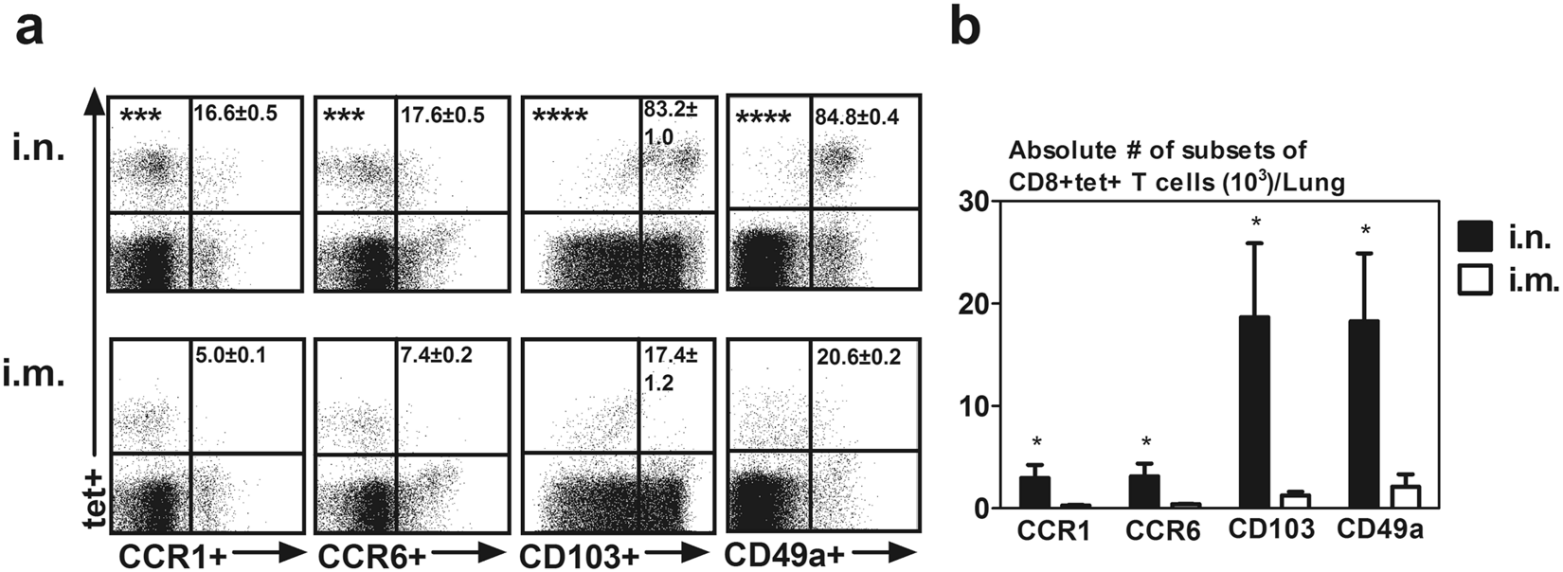
Protein expression of T_RM_ surface markers by replication-defective viral-vectored respiratory mucosal immunization-induced Ag-specific CD8 T cells in the lung. Lung mononuclear cells from mice immunized with viral vector vaccine via either respiratory mucosal (i.n.) or parenteral (i.m.) route for four weeks were immunostained for surface markers CCR1, CCR6, CD103 and CD49a and analyzed using flow cytometry. (**a**) Representative dot plots showing frequencies of tet+CCR1, tet+CCR6+, tet+CD103+, tet+CD49a+CD8 T cells out of total CD8+tet+T cells in the lung of i.n. and i.m. immunized mice. (**b**) Bar graph showing absolute numbers of tet+CCR1, tet+CCR6+, tet+CD103+, tet+CD49a+CD8 T cells in the lung of i.n. and i.m. immunized mice. Data are presented as mean ± S.E.M. of three mice per group, representative of three independent experiments. *P < 0.05, ***P < 0.001, ****P < 0.0001 compared with i.m. immunization.

Based on their unique gene expression profile and differential localities in the lung, we next selected to determine protein expression levels of CCR1, CCR6, CD103 (*itage*) and CD49a (*itga1*or VLA-1) on respiratory mucosal immunization-induced Ag-specific memory CD8 T cells at 4 weeks post-immunization. Although some genes such as *Cxcr5* and *Ccr7* were also increased in these cells, they were not included in our protein expression analysis as they pertains more to the homing of T cells to secondary lymphoid organs^32^. Nor was CCR8 protein examined due to limited murine immunoreagents. By flow cytometry only a smaller frequency of CD8+tet+T cells (~20%) expressed CCR1 and CCR6 protein in the lung of i.n. immunized animals (Fig. 2a). In sharp contrast, >80% of Ag-specific CD8 T cells expressed T_RM_ surface markers CD103 and CD49a (VLA-1) (Fig. 2a). In consistent with increased frequencies, we also observed significantly higher numbers of Ag-specific CD8 T cells expressing CD103 or CD49a than those expressing CCR1 or CCR6 in the lung (Fig. 2a). In comparison, very few Ag-specific memory CD8 T cells induced by i.m. immunization expressed T_RM_ surface markers CD103 and CD49a (VLA-1). Together, these data demonstrate that respiratory mucosal TB immunization generates Ag-specific T cells with typical properties of T_RM_ cells in the lung.

### Viral-vectored respiratory mucosal immunization-induced Ag-specific CD8 T cells acquires VLA-1 expression in the draining lymph node

Having established that the majority of respiratory mucosal TB immunization-induced lung tissue Ag-specific CD8 T cells express classical resident memory surface markers, CD103 and CD49a^5^, we sought to systematically examine the kinetic expression of these T_RM_ surface markers during various phases (effector/expansion, contraction and memory) of T cell responses following respiratory mucosal immunization. To this end we first characterized the phases of T cell responses following viral vector immunization. The CD8+tet+T cells in the lung significantly increased at 10 days and peaked at 14 days post-respiratory mucosal immunization, consistent with the effector/expansion phase of T cell responses (Supplementary Fig. 1). Between 14 and 28 days, the number of CD8+tet+T cells markedly decreased by more than 80% from the peak time indicative of the contraction phase of T cell responses. From 28 days until 45 days, the number of CD8+tet+T cells in the lung remained stable, hence being in the memory phase of T cell responses. In comparison, parenteral intramuscular (i.m.) immunization led to much smaller levels of Ag-specific T cell responses in the lung in various phases (Supplementary Fig. 1).

**Figure 3:**
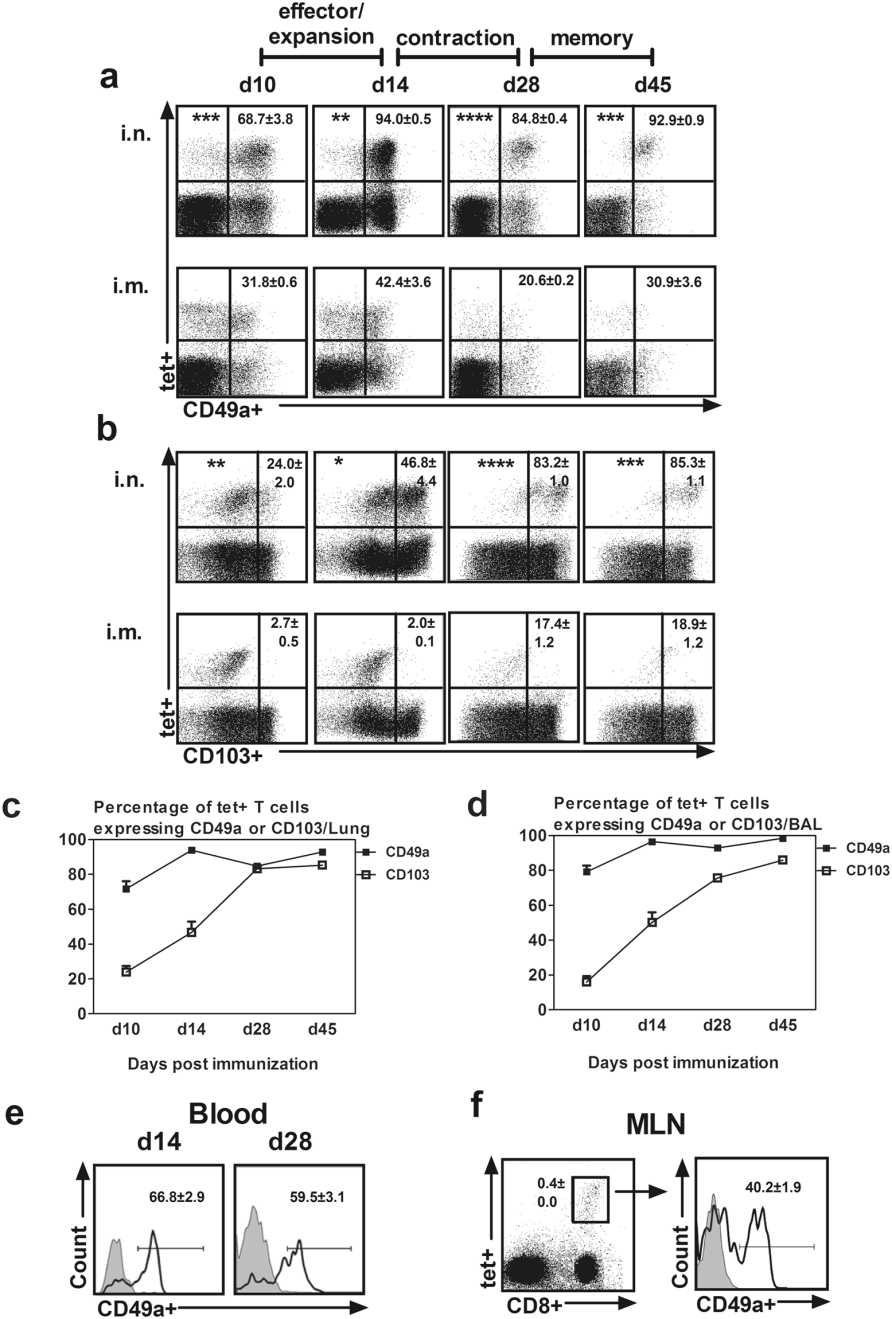
Expression of T_RM_ surface markers on replication-defective viral-vectored respiratory mucosal immunization-induced Ag-specific CD8 T cells in different phases of T cell responses. Mononuclear cells from lung, BAL, peripheral blood and mediastinal lymph nodes (MLN) obtained at designated time points post-immunization were immunostained for CD49a and CD103 and analyzed using flow cytometry. (**a/b**) Representative dot plots showing frequencies of tet+ CD49a+ and tet+ CD103+ CD8 T cells out of total CD8+tet+T cells in the lung of respiratory mucosal (i.n.) and parenteral (i.m.) immunized mice in the effector/expansion phase (d10/d14), contraction phase (d14/d28) and memory phase (d28/d45) of T cell responses. (**c**) Line graph comparing kinetic changes in the expression of CD49a and CD103 on Ag-specific CD8 T cells in the lung induced by viral vector respiratory mucosal immunization. (**d**) Line graph comparing kinetic changes in the expression of CD49a and CD103 on Ag-specific CD8 T cells in the bronchoalveolar lavage fluid (BAL) induced by respiratory mucosal immunization. (**e**) Representative histograms showing frequencies of CD8+tet+T cells expressing CD49a in the blood at d14 and d28 post- viral vector respiratory mucosal immunization. (**f**) Representative dot plot showing the frequency of CD8+tet+T cells out of total T cells in MLN at d14 post- viral vector respiratory mucosal immunization, and the representative histogram showing the frequency of CD8+tet+T cells expressing CD49a. Data are presented as mean ± S.E.M. of three mice per group per time point, representative of three independent experiments. *P < 0.05, **P < 0.01, ***P < 0.001, ****P < 0.0001 compared with i.m. immunization.

We next examined CD103 and CD49a expression on Ag-specific CD8 T cells in the lung in different phases of T cell responses. The majority of CD8+tet+T cells in the lung of i.n. immunized animals expressed CD49a upon arrival at the lung and in the expansion/effector phase (d10–d14) and became further enriched for CD49a expression in the contraction (d14–d28) and memory phases (d28–d45) (Fig. 3a/c **)**. In contrast to CD49a, only a small frequency of CD8+tet+T cells (24% at d10) expressed CD103 in the expansion/effector phase and it progressively increased over the contraction and memory phases (46% at d14 up to 85% at d45) (Fig. 3b/c). The expression profile of CD49a and CD103 on airway luminal (BAL) CD8+tet+T cells was identical to that of the lung cells. Upon closer examination, from d28 onward the majority of CD8+tet+T cells in the lung and airway lumen co-expressed both CD49a and CD103 indicating the acquisition of a bona fide T_RM_ property (Supplementary Fig. 2a/b). In comparison, parenteral intramuscular (i.m.) immunization-induced Ag-specific CD8 T cells only temporarily expressed CD49a and mostly lacked CD103 expression in different phases of T cell responses (Fig. 3a/b). These data suggest that following respiratory mucosal TB immunization the Ag-specific CD8 T cells acquired CD49a (VLA-1) expression before their arrival at the lung whereas they acquired CD103 expression after they entered the lung.

To determine the geographical origin of CD49a acquisition, we examined the CD49a expression on CD8+tet+T cells in the circulation and mediastinal lymph node (MLN), the draining lymph node of the lung. Indeed, a significant number of CD8+tet+T cells in the blood expressed CD49a in the effector phase of T cell responses (d14) (Fig. 3e), consistent with its marked expression on such T cells primed in MLN (d14) (Fig. 3f). Circulating CD8+tet+T cells continued to show high levels of CD49a expression in the memory phase (d28) (Fig. 3e). These data suggest that although respiratory mucosal TB immunization-induced Ag-specific T cells in the lung co-express both CD49a and CD103, these T_RM_ markers are acquired in distinct tissue sites with CD49a (VLA-1) expressed on respiratory mucosal vaccine-induced T cells even before they home to the lung.

### VLA-1 is not required for trafficking of viral-vectored respiratory mucosal immunization-induced Ag-specific CD8 T cells to the lung

**Figure 4:**
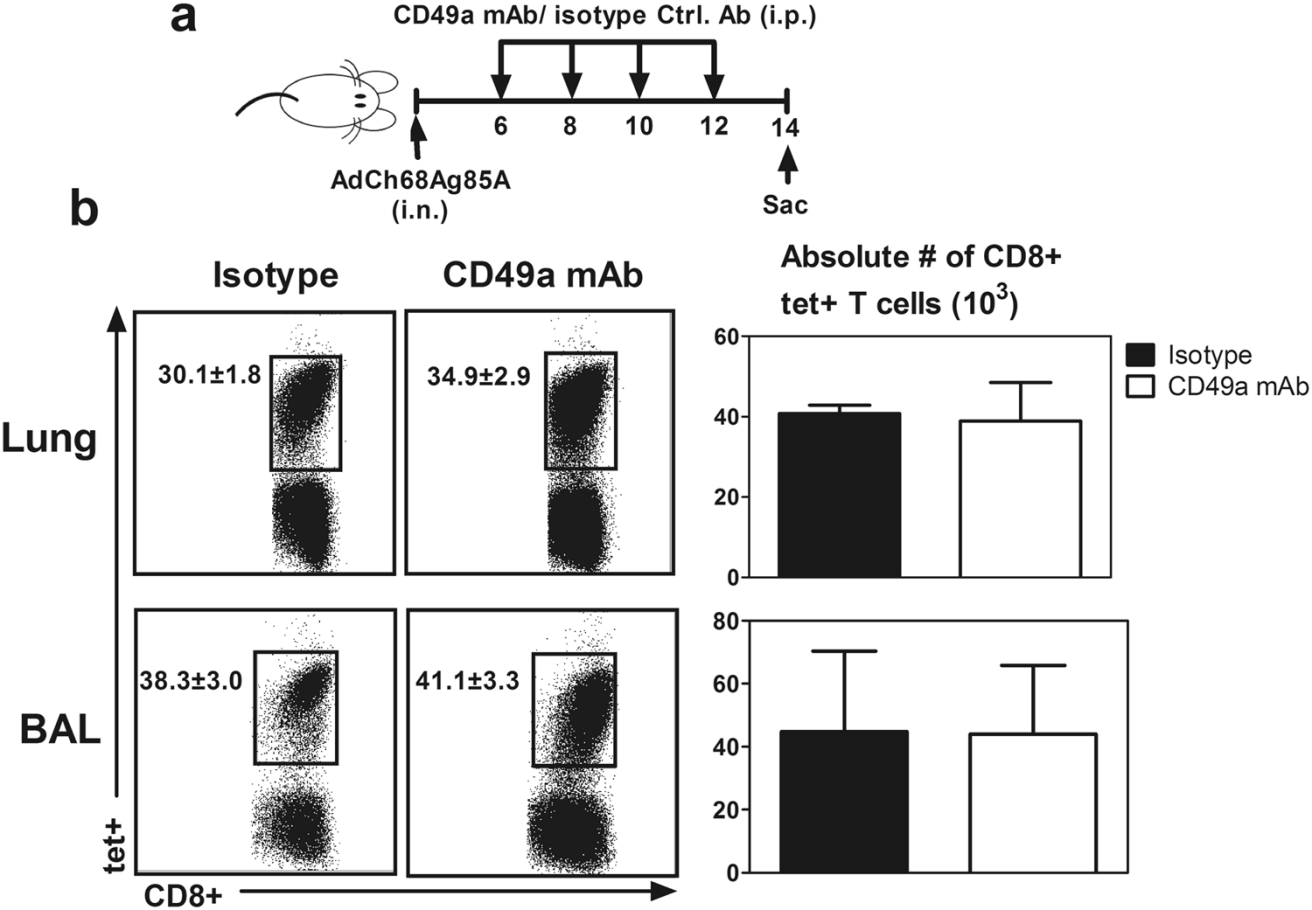
Role of VLA-1 in trafficking of replication-defective viral vector respiratory mucosal immunization-induced Ag-specific CD8 T cells to the lung during the effector phase of T cell responses. (**a**) Experimental schema depicting the timing of administration of CD49a blocking mAb and isotype control antibody. Blocking/isotype antibodies were administered 6–12 days post- viral vector respiratory mucosal immunization when Ag-specific tet+ CD8 T cells clonally expanded in the draining lymph nodes and in the process of homing to the lung. (**b**) Representative dot plots showing frequencies of CD8+tet+T cells out of total CD8 T cells in the lung and bronchoalveolar lavage fluid (BAL) of the control and CD49a-blocked mice. Bar graphs comparing absolute numbers of Ag-specific tet+ CD8 T cells in the lung and BAL. Data are presented as mean ± S.E.M. of three mice per group from one experiment.

Having demonstrated that the prominent CD49a expression on Ag-specific CD8 T cells outside and within the lung, we postulated that VLA-1 played a role in the trafficking of Ag-specific CD8 T cells to the lung. To address this question, we blocked CD49a during the initial stage of T cell activation (d6–d12) before Ag-specific CD8 T cells arrived *en masse* at the lung (Fig. 4a) by using a well-established CD49a functional blocking antibody (CD49a mAb) delivered via intraperitoneal route^33^. Analysis of CD49a expression on Ag-specific CD8 T cells in the MLN, blood, lung and BAL confirmed complete blockade of CD49a receptor following delivery of CD49a mAb but the isotype control antibody had no effect (Supplementary Fig. 3). Of interest, CD49a blockade did not change the recruitment of CD8+tet+T cells to the lung and airway lumen during the effector/expansion phase (Fig. 4b). These data suggest that VLA-1 does not play a significant role in T cell trafficking to the lung mucosal sites during the initial phase of T cell activation following viral vector mediated respiratory mucosal TB immunization.

### VLA-1 negatively regulates viral-vectored vaccine-induced Ag-specific CD8 T cells during the contraction phase in the lung

**Figure 5:**
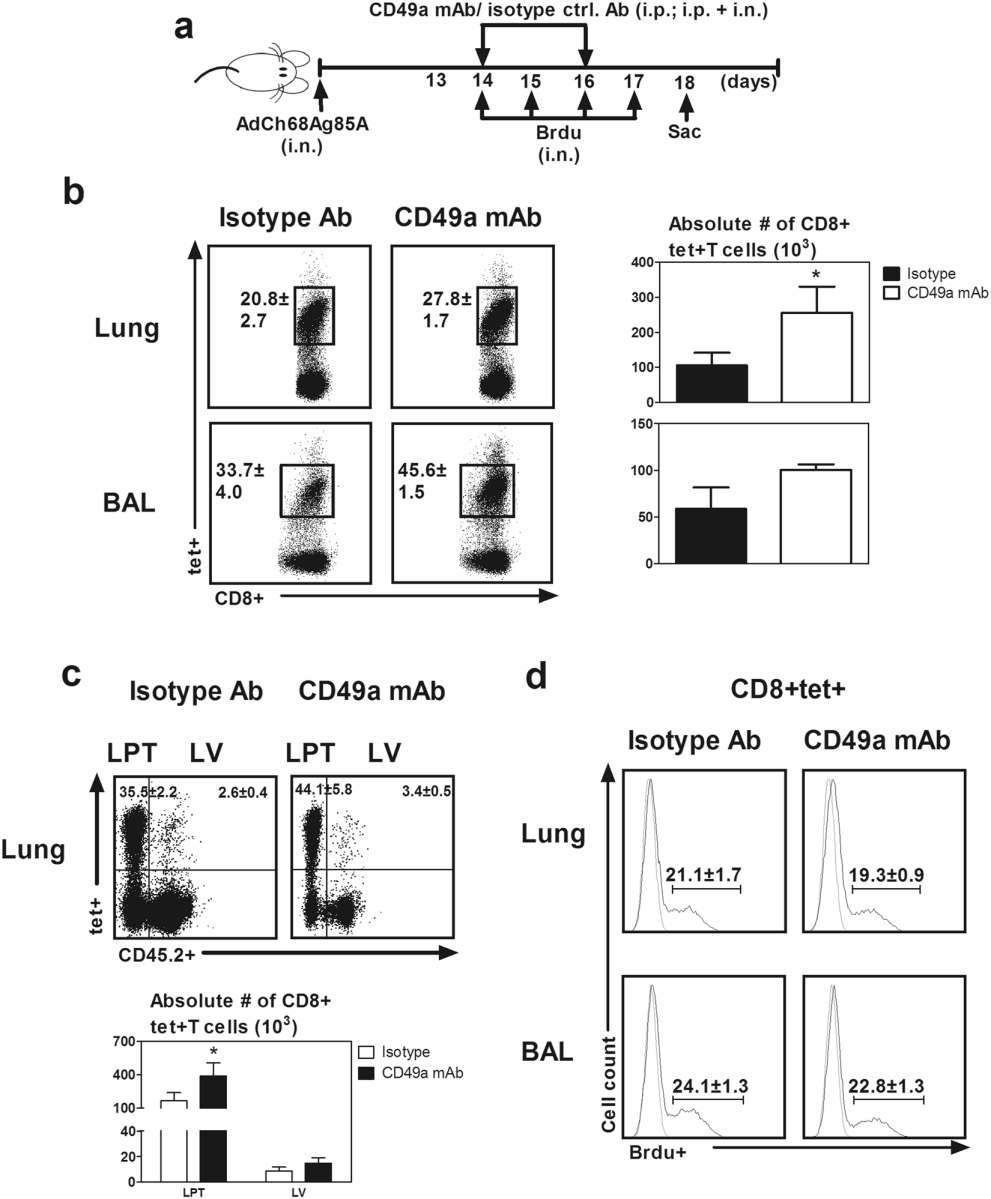
Role of VLA-1 in regulation of replication-defective viral-vectored respiratory mucosal immunization-induced Ag-specific CD8 T cells in the lung during the contraction phase of T cell responses. (**a**) Experimental schema depicting the timing of administration of CD49a blocking mAb and isotype control antibody. Blocking and isotype control antibodies were administered at d14 and d16 post- viral vector respiratory mucosal immunization when Ag-specific CD8 T cells in the lung sharply declined. Mice were sacrificed 3 min after i.v. injection of fluorochrome conjugated CD45.2 mAb to differentiate T cells in the lung vasculature from those located in the lung parenchyma. In separate experiments, the mice were treated as above and bromodeoxyuridine (BrdU) was administered i.n. for consecutive four days to assay the *in vivo* proliferation rate of Ag-specific CD8 T cells. (**b**) Representative dot plots showing frequencies of CD8+tet+T cells out of total CD8 T cells in the lung and bronchoalveolar lavage fluid (BAL). Bar graphs comparing absolute numbers of Ag-specific tet+ CD8 T cells in the lung and BAL between isotype control antibody and CD49a mAb treated mice. (**c**) Representative dotplots showing frequencies of CD8+tet+T cells out of total CD8 T cells in the lung parenchymal tissue (LPT) and lung vasculature (LV). Bar graphs comparing absolute numbers of Ag-specific CD8+tet+T cells in the LPT and LV between isotype control antibody and CD49a mAb treated mice. (**d**) Representative histograms comparing frequencies of BrdU+ proliferating Ag-specific CD8 T cells in lung and BAL between isotype control antiobody and CD49a mAb treated mice. Data are presented as mean ± S.E.M. of three mice per group, representative of two independent experiments. *P < 0.05, compared to isotype control group.

Integrins such as VLA-1 have previously been implicated in intracellular signalling pathways to regulate cell survival and cell death^9, 10^ which may be involved in the contraction phase of T cell responses. To determine whether VLA-1 was involved in regulating the contraction of antigen-specific T cells following their effector/expansion responses in the lung, immunized mice were treated with CD49a mAb starting from day 14 post-immunization to block VLA-1 pathway and CD8 + tet+ T cells were examined without Ag re-stimulation at day 18 (Fig. 5a). CD49a blockade led to increased frequencies of CD8+tet+T cells both in the lung and airway (Fig. 5b). It also led to 2–3 times more CD8+tet+T cells in the lung, compared to the isotype control (Fig. 5b). Using an intravascular staining protocol we found that the rise in CD8+tet+T cells in the lung of anti-VLA-1 treated animals occurred only in the lung parenchymal tissue (LPT) while the CD8+tet+T cells in the lung vasculature (LV) remained comparable in numbers to isotype Ab treated animals (Fig. 5c). This finding suggests that VLA-1negatively regulates only the CD8+tet+T cells in the LPT but not those in the LV during the contraction phase.

That VLA-1 blockade increases the number of vaccine-activated CD8+tet+T cell during the contraction phase raised the question whether VLA-1 affects T cell contraction via regulating T cell proliferation. To test this possibility, in separate experiments we examined the proliferation rate of Ag-specific CD8 T cells with and without CD49a blockade by using an *in vivo* BrdU incorporation assay. T cell BrdU labeling was accomplished following repeated intranasal deliveries of BrdU (Fig. 5a). We found the rates of CD8+tet+T cell proliferation in the lung and BAL of animals with CD49a blockade (CD49a mAb) were comparable to isotype controls (Fig. 5d), suggesting that VLA-1 impacts T cell contraction independent of regulation of T cell proliferation.

**Figure 6:**
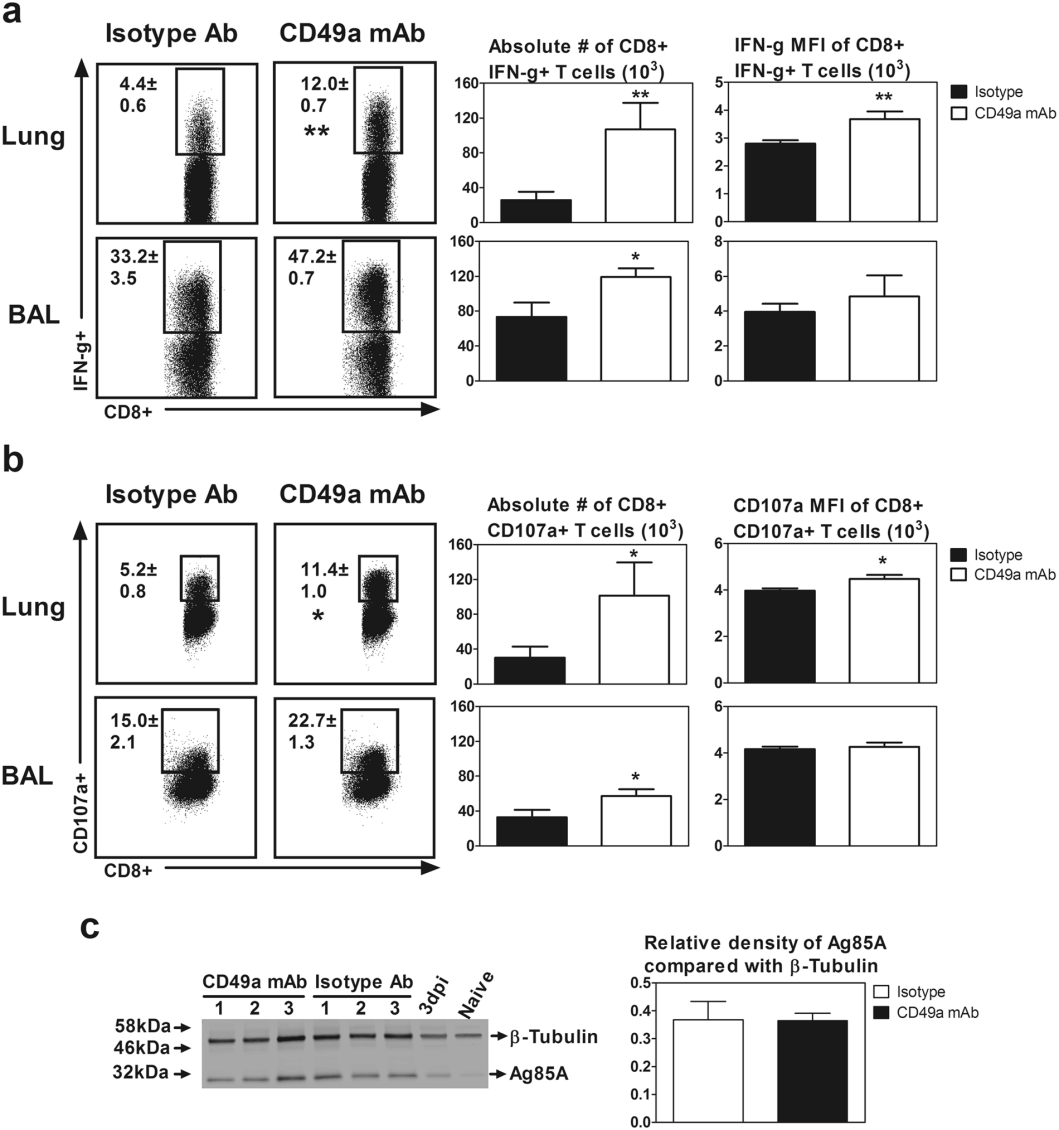
Role of VLA-1 in regulation of effector functions of replication-defective viral-vectored respiratory mucosal immunization-induced Ag-specific CD8 T cells in the lung during the contraction phase of T cell responses. Experimental conditions were described in Fig. 5a except that the cells were *ex vivo* re-stimulated with Ag85A antigens. (**a**) Representative dot plots showing frequencies of CD8+ IFN-γ+ T cells out of total CD8 T cells in the lung and BAL. Bar graphs comparing absolute numbers and IFN-γ mean fluorescence intensity (MFI) of CD8+ IFN-γ+ T cells in the lung and bronchoalveolar lavage fluid (BAL) between isotype control antibody and CD49a mAb treated mice. (**b**) Representative dot plots showing frequencies of CD8+ CD107a+ degranulating T cells out of total CD8 T cells in the lung and BAL. Bar graphs comparing absolute numbers and CD107a MFI of CD8+ CD107a+ T cells in the lung and BAL between isotype control antibody and CD49a mAb treated mice. (**c**) Western blot depicts levels of Ag85A protein and β-Tubulin in the total lung of 3 mice (1–3) treated with anti-CD49a mAb, 3 mice (1–3) treated with isotype antibody, a mouse immunized for 3 days (3dpi) and an unimmunized mouse (Naïve). Bar graph shows relative levels of Ag85A calculated in respect to β-Tubulin using Image Studio Lite. Data are presented as mean ± S.E.M. of three mice per group, representative of two independent experiments except western plot data, which is representation of one experiment. *P < 0.05, **P < 0.01 compared to isotype control group.

Apart from tetramer specificity and proliferation of CD8+tet+T cell following CD49a blockade, we further examined other functional properties of CD8 T cells including IFN-γ production and degranulation as indicated by CD107a expression upon *ex vivo* Ag re-stimulation. Indeed, compared to their control counterparts, *in vivo* CD49a blockade led to significantly increased frequencies and numbers of Ag-specific CD8 T cells capable of IFN-γ production (Fig. 6a) and degranulation (Fig. 6b) upon *ex vivo* Ag re-stimulation. Furthermore, we found that *in vivo* CD49a blockade also led to significantly increased production of IFN-γ and degranulation marker CD107a per cell basis measured by mean fluorescent intensity (MFI) of signals (Fig. 6a/b).

That VLA-1 blockade increased the number of vaccine-activated CD8+tet+T cell during the contraction phase also raised the question whether VLA-1 affects T cell contraction via altering the viral clearance as such affecting the level of Ag85A protein in the lung. Thus, in a separate experiment we quantified the antigenic (Ag85A) load in the lung following immunization in isotype and CD49a blocking antibody treated animals (Fig. 5a). Mouse either left without immunization (naïve) or immunized and sacrificed 3 days post-immunization was used as negative and positive controls, respectively. Western blot analysis of the total lung protein showed comparable levels of Ag85A protein in isotype and CD49a blocking antibody treated animals, suggesting that the differential contraction level of CD8+tet+T cell in CD49a blocking antibody treated animals is independent of antigenic load in the lung (Fig. 6c).

Taken together these data suggest that VLA-1 pathway plays a critical role in negatively regulating Ag-specific CD8 T cell responses during the contraction phase following viral vector based respiratory mucosal TB immunization.

### VLA-1 is not required for maintenance of viral-vectored respiratory mucosal immunization-induced Ag-specific T_RM_ during the memory phase in the lung

**Figure 7:**
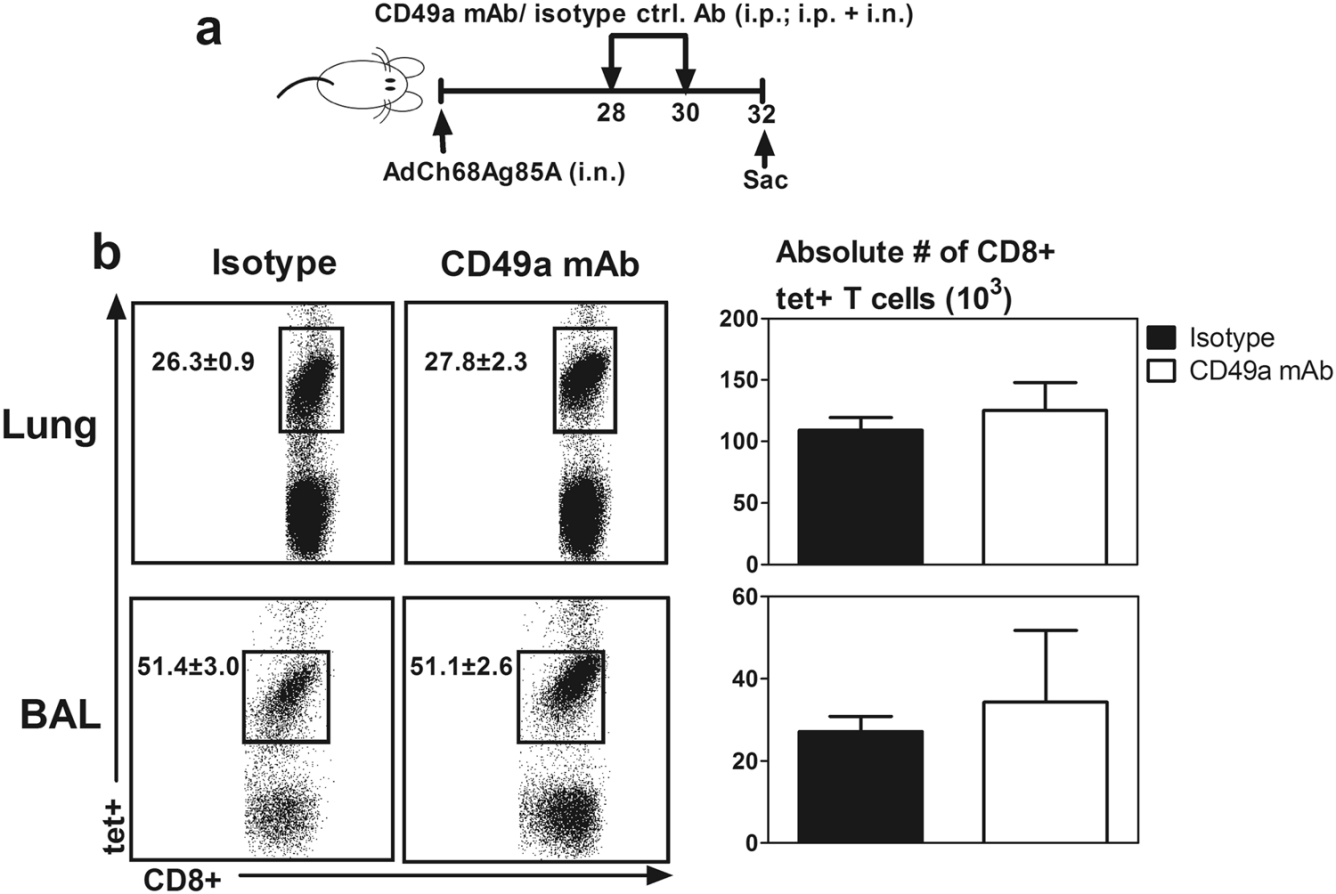
Role of VLA-1 in maintenance of replication-defective viral-vectored respiratory mucosal immunization-induced Ag-specific T_RM_ cells in the lung during the memory phase of T cell responses. (**a**) Experimental schema depicting the timing of administration of CD49a blocking mAb and isotype control antibody. Blocking and isotype control antibodies were administered at d28 and d30 post- viral vector respiratory mucosal immunization after the development of Ag-specific memory CD8 T cells in the lung. (**b**) Representative dot plots showing frequencies of Ag-specific tet+ CD8 T cells out of total CD8 T cells in the lung and bronchoalveolar lavage fluid (BAL). Bar graphs comparing absolute numbers of CD8+tet+T cells in the lung and BAL between isotype antibody and CD49a mAb treated mice. Data are presented as mean ± S.E.M. of three mice per group, representative of two independent experiments.

VLA-1 was previously shown to contribute to the retention of Ag-specific CD8 T cells in the lung tissue after influenza infection^12^. We have shown that immune protective Ag-specific CD8 T cells induced by viral vector respiratory mucosal immunization persist in the lung for a long time^34, 35^ and we have here found these cells to be of T_RM_ phenotype (Fig. 2/3). We thus next determined whether VLA-1 also played a role in maintenance of viral vector vaccine-induced T_RM_ cells in the lung. To test this, CD49a receptor was functionally blocked for a total of four days beginning from day 28 of the memory phase following respiratory mucosal immunization and CD8+tet+T cells were examined at day 32 (Fig. 7a). We found that CD49a blockade during the memory phase had no effect on both the frequencies and numbers of CD8+tet+T cells in the lung and airway, compared to the isotype control animals (Fig. 7b). These data suggest that VLA-1 is not required for the retention of Ag-specific tissue resident memory CD8 T cells in the lung following replication-defective viral vector respiratory mucosal TB immunization.

## Discussion

Effective vaccines for infectious diseases such as tuberculosis, the leading cause of global morbidity and mortality, are currently lacking^36^. Protection against these diseases relies on T cell immunity and the induction and maintenance of tissue resident memory T cells (T_RM_) at the site of infection is critical for vaccine-induced protection^2, 3, 37^. Improved knowledge in the expression and roles of T_RM_ surface integrin molecules following vaccination will help develop effective vaccination strategies against infections at mucosal sites of pathogen entry. Here we show that a replication-defective virus-vectored TB vaccine administered via the respiratory mucosal route induces Ag-specific T_RM_ in the lung characterized by high-level expression of T_RM_ surface markers VLA-1 and CD103. Of importance, our study shows that expression of these surface markers is differentially regulated post-respiratory mucosal vaccination. VLA-1 is initially markedly upregulated on T cells during the effector/expansion phase in the mediastinal lymph node draining the site of mucosal vaccination and remains highly expressed after the arrival of these T cells in the lung through both the contraction and memory phases. In comparison, CD103 expression is acquired by T cells primarily after they were primed in the draining lymph nodes and trafficked into the lung. However, we find that despite its acquisition outside the lung, VLA-1 is not required for the trafficking of circulating Ag-specific CD8 T cells to the lung mucosa. We further find that VLA-1 plays a role in the contraction phase in the lung by negatively regulating T cell responses while it does not play a significant role in the maintenance of T_RM_ during the memory phase. Our study provides new information on vaccine-inducible T_RM_ in the lung and shall help develop effective respiratory mucosal vaccination strategies against pulmonary TB.

Replication-defective viral vectored vaccines are attractive vaccine carriers, particularly for respiratory mucosal immunization strategies given their potency and safety^18^. We showed previously that replication-defective adenoviral vector tuberculosis vaccine, AdAg85A, a model viral vector vaccine used in the current study, when administered via respiratory mucosal route, induced long lasting, protective Ag-specific CD8 T cells in the lung and airway^21, 34, 38^. Here we demonstrate that these cells preferentially express classical T_RM_ surface markers VLA-1 and CD103, which is in line with the phenotype of T_RM_ induced by respiratory viral pathogen exposure^12, 39, 40^. However, in contrast to viral pathogen-specific CD8 T_RM_ counterparts that mostly acquire VLA-1 expression within the local lung microenvironment immediately after peak T cell responses^12^, we find VLA-1 to be expressed on Ag-specific CD8 T cells upon their activation in the dLN following replication-defective viral vector respiratory mucosal vaccination. This difference is likely attributed to differential inflammatory signals and antigenic persistence resulting from replicating viral pathogen exposure versus attenuated replication-defective viral vector vaccination. In support of such difference in immunologic sequela, it has been observed that while continuing peripheral T cell supply contributes to the maintenance of influenza-specific CD8 T cells in the lung^41^, *in-situ* T cell proliferation alone, in the absence of peripheral supply, maintains Ag-specific CD8 T cells in an Ag-dependent manner following replication-defective viral vector respiratory mucosal vaccination^34^.

Our finding that VLA-1 is not involved in the initial trafficking of replication-defective viral vector vaccine-activated circulating CD8 T cells to the lung mucosa during the effector phase of T cell responses is in accord with the previous observation in a model of influenza viral infection^12^. These findings together are at odds with the findings from the models of rheumatoid arthritis, delayed type hypersensitivity (DTH)^33^ and cancer^42^ where VLA-1 is seen to play a role in T cell recruitment to the peripheral tissue sites. These observations suggest that VLA-1 is differentially required in T cell trafficking, depending on the specific tissue site and immunologic tissue microenvironment.

So far there have not been any studies to examine the role of VLA-1 in the contraction phase of T cell responses. Here we show VLA-1 to negatively regulate replication-defective viral vector vaccine-induced Ag-specific CD8 T cells during the contraction phase in the lung. Blockade of VLA-1 in our model significantly slows down the pace of T cell contraction in the lung following the initial effector/expansion responses of the T cells, resulting in increased numbers and activation of T cells. Although it remains unclear how VLA-1 negatively regulates the contraction of T cells destined to become T_RM_, it is likely accomplished via its roles in regulating the survival and apoptosis^13^, resources competition^43, 44^, and differentiation^45^ of Ag-specific T cells. We further show here that although the Ag-specific CD8 T_RM_ following the contraction phase continue to express VLA-1, VLA-1 does not seem to play a significant role in maintaining Ag-specific CD8 T_RM_ during the memory phase. Our finding contrasts the finding from the model of influenza viral exposure where VLA-1 blockade resulted in the loss of Ag-specific memory CD8 T cells in the lung^12^. It is likely that in our replication-defective viral vector model, VLA-1 deficiency may well be compensated for by the function of other T_RM_ surface integrin molecules such as CD103, which remains highly expressed on our replication-defective viral vector vaccine-induced T_RM_. Indeed, CD103 was previously shown, via its interaction with E-cadherin on epithelial cells, to potentiate the retention of CD8 T cells in the lung^46^.

Lack of biological correlates of immune protection is a major bottleneck for development of new prophylactic and immunotherapeutic vaccines for infectious diseases such as TB^47, 48^. As such, the protective efficacy of a new vaccine in question remains unknown until the completion of costly late-phase clinical efficacy trials. In this regard, generation of T_RM_ in the lung is a reliable biological correlate for protection following respiratory mucosal TB immunization^49^. However, it may be difficult to access and analyze T_RM_ in human lungs. One possible way is to identify biological correlates of successful induction of lung immunity on T cells in the circulation. However, circulating Ag-specific CD8 T cells constitute a small fraction of total CD8 T cells. Our data imply that VLA-1, as opposed to CD103, expressed by the majority of circulating vaccine-induced Ag-specific CD8 T cells, may serve as a surrogate marker to reliably predict T_RM_ generation at the respiratory mucosa. This is in contrast to low number of VLA-1-expressing CD8+tet+T cells found in blood following replication-defective viral vector intramuscular vaccination (Supplementary Fig. 4). It has long been believed that mucosal homing molecules would be potential biological correlates of protective immunity as they are related to the T cell subsets that likely have the ability to populate mucosal sites. As such, integrin molecules CD103^50^ and VLA-4^51^ were identified as surrogate markers in the blood to predict T cell responses in the female genital tract and respiratory mucosa, respectively. Our data suggest that not only the mucosal homing molecules but also the molecules that are programmed to express on activated T cells during lineage differentiation in draining lymph nodes may also be considered for the screening of biological correlates of protective immunity. However, the finding in murine models needs cautious interpretation in humans due to disparity in subsets of T cells responding to viral vectored vaccines^52, 53^.

In conclusion, our current study has examined the kinetic expression of classic T_RM_ markers VLA-1 and CD103 and further deciphered the role of VLA-1 integrin, in different phases of CD8 T cell responses following respiratory mucosal vaccination with a replication-defective viral vector TB vaccine. We find VLA-1 to be expressed on viral vector vaccine-induced CD8 T cells before and after they trafficked to the lung and to play a differential role in various phases of T cell responses. These findings hold implications in understanding vaccine-inducible T_RM_ in the lung and developing novel vaccination strategies against respiratory infectious diseases such as TB.

## Material and Methods

### Ethics approval

All animal experiments in this study were approved by the animal research ethics board of McMaster University, and were performed in accordance with the approved guidelines for animal experimentation at McMaster University.

### Animals

Female BALB/c 6 to 8 weeks old mice were purchased from Charles River Laboratories (Charles River, St Constant, Quebec, Canada) and housed in specific pathogen-free Level B rooms within the central animal facility at McMaster University.

### Immunization with viral vectored vaccine

A replication-deficient adenovirus expressing immunodominant *Mycobacterium tuberculosis* antigen Ag85A was used to immunize animals via either respiratory mucosal or parenteral route. Respiratory mucosal route of immunization was carried out by intranasal (i.n.) delivery of 1 × 10^7^ plaque forming unites (pfu) per mouse in 25 μl of total volume^21^. In some experiments, mice were immunized intramuscularly (i.m.) via quadriceps muscles with the same dose of the vaccine in 100 μl of total volume as previously described^38^.

### Bronchoalveolar lavage, lung, blood and lymph node mononuclear cell isolation

After anesthetizing animals, peripheral blood was collected from abdominal artery in tubes containing 300 μl of Heparin (40 unites/ml) (Sigma-Aldrich, St Louis, MO, USA). Then, mice were sacrificed by exsanguination. Airway luminal cells were collected through bronchoalveolar lavage (BAL)^21^. Lung mononuclear cells were isolated from perfused lungs as previously described^38^. Lymph nodes were crushed using frosted glass slides, then single-cell suspension was obtained by crushing the organ through 40μm basket filter. Heparinized blood samples were treated twice with ACK lysis buffer (Invitrogen, Burlington, ON, Canada) to remove all red blood cells and washed with phosphate-buffered saline. All isolated cells were resuspended in RPMI 1640 medium supplemented with 10% fetal bovine serum, 1% penicillin–streptomycin, and 1% L-glutamine.

### Sorting of Ag-specific CD8 T cells and PCR-array gene expression analysis

Ag-specific CD8 T cells were sorted from lung mononuclear cells using flow sorter. Briefly, lung mononuclear cells were immunostained with a tetramer specific for the Ag85A CD8 T cell peptide (MPVGGQSSF) bound to BALB/c MHC class I allele H-2L^d^ (National Institutes of Health, Tetramer Core, Atlanta, GA, USA) for 1 h in the dark at RT. Immunostained cells were then sorted using BD FACS Aria III cell sorter (BD Pharmingen, San Jose, CA, USA). In addition, CD8 T cells were isolated from naïve lung mononuclear cells using mouse CD8a (Ly-2) microbeads (Miltenyi Biotec Inc., Auburn, CA, USA). Total mRNA of the purified cells was extracted using RNeasy kit (Qiagen, Toronto, ON, Canada) and then converted to cDNA using an RT^2^ First Strand Kit (Qiagen, Toronto, ON, Canada). cDNAs were added to RT^2^ qPCR master mix and then the mixture was aliquoted across a custom made mouse RT^2^ profiler PCR-array profiling the expression of handpicked genes encoding chemokine receptors, adhesion molecules and T cell effector/cell surface molecules (Supplementary Table 1) (Qiagen, Toronto, ON, Canada). PCR reactions were conducted using a 7900HT fast real-time PCR system with fast 96-well block module (Life Technologies Inc., Burlington, ON, Canada). For data analysis, relative gene expressions with at least 2-fold changes (P < 0.05) were considered to be significant.

### Cell stimulation, immunostaining, and flow cytometry

The isolated mononuclear cells were seeded in U-bottom 96-well plates at a concentration of 20 million cells/ml for lungs and lymph nodes, 10 million cells/ml for blood, and 5 million cells/ml for BAL. Ag-specific T cells were identified using a tetramer specific for the Ag85A CD8 T cell peptide (MPVGGQSSF) bound to BALB/c MHC class I allele H-2Ld and Phycoerythrin fluorochrome (National Institutes of Health Tetramer Core, Atlanta, GA, USA) for 1 h in the dark at RT^25, 54^. For intracellular cytokine staining and cytotoxicity assay using a degranulation marker CD107a^55^, mononuclear cells were incubated at 37 °C in the presence of Golgi plug (5 mg/ml brefeldin A; BD Pharmingen, San Jose, CA, USA), Golgi stop (0.26% (w/w) Monensin; BD Pharmingen, San Jose, CA, USA), and FITC-conjugated CD107a monoclonal antibody (mAb) (clone 1D4B) (1:50) (Biolegend, San Diego, CA, USA) simultaneously with or without stimulation with an immunodominant *M.tb* antigen 85 A (Ag85A) specific CD8 T cell peptide (MPVGGQSSF) at a concentration of 1 µg/well for 5–6 h. Incubation was followed by washing and blocking using CD16/CD32 block Ab (clone 2.4G2) (1:150) (BD Pharmingen, San Jose, CA, USA) in 0.5% bovine serum albumin/PBS for 15 min on ice. Cells were then washed and stained using cell surface mAbs. Then, cells were washed, permeabilized, and stained intracellularly. For some experiments, only tetramer and extracellular staining were carried out without incubation and antigen stimulation, and cells then were fixed using 1% paraformaldehyde/PBS at RT for 10–15 min. The fluorochrome-conjugated mAbs used included CD3-V450 (clone 17A2) (1:200), CD8a-PE-Cy7 (clone 53–6.7) (1:400), CD4-APC-Cy7 (clone RM4–5) (1:400), CD49a (α1 domain of VLA-1)-Alexa Fluor 647 (clone Ha31/8) (1:100), CD103-Biotin (clone 2E7) (1:100) (Qdot-800-Streptavidin (1:500)), IFN-γ-PerCP-Cy5.5 (clone XMG1.2) (1:200) (BD Pharmingen, San Jose, CA, USA), CCR1-APC (clone 643854) (1:50), and CCR6- Alexa Fluor 488 (clone 140706) (1:100) (R&D system, Minneapolis, MN, USA). Immunostained cells were run on an LSR II flow cytometer (BD Biosciences, San Jose, CA, USA) and analyzed using FlowJo software (version 10; Tree Star, Ashland, OR, USA).

### Intravascular immunostaining

Intravascular immunostaining was carried out as previously described^29^. Briefly monoclonal anti-CD45.2-Alexa Fluor 700 mAb (clone 104) (BD Pharmingen, San Jose, CA, USA) was prepared at 1 µg in 250 µl concentration and injected intravenously via tail vein. Within three minutes after injection, animals were sacrificed, and blood, BAL and lung were obtained for analysis.

### *In vivo* VLA-1 blockade

To investigate the role of VLA-1 *in vivo*, we used the function blocking hamster mAb (clone Ha31/8) against CD49a and the hamster isotype-matched control Ab (clone Ha4/8) (BD Pharmingen, San Jose, CA, USA) for blocking experiments. For each injection, the antibodies were administered intraperitoneally (i.p.) (150 µg or 200 µg/mouse) or intranasally (i.n.) (50 µg/mouse)^33^.

### 5-Bromo-2’-deoxyuridine (BrdU) administration for evaluation of *in vivo* T cell proliferation

Frequency of proliferating Ag–specific CD8 T cells in the lung was determined by *in vivo* Brdu incorporation assay as previously described^34^.

### Western blot analysis of Ag85A protein in the lung

The lungs from naïve mice or i.n immunized mice with or without treatment with blocking or isotype control antibodies were collected and perfused by injecting cold Hank’s buffer through the right ventricle in order to remove intravascular mononuclear cells, and kept in cold Hank’s buffer. Total lung protein was subjected to western blotting with an anti-Ag85A monoclonal antibody (clone TD-17) and anti-β-Tubulin monoclonal antibody (clone TUB2.1) (Sigma-Aldrich, St. Louis, MO, USA) as a control. Densitometric quantitation of western blot analysis was conducted using Image Studio Lite (LI-COR Biosciences, Lincoln, NE, USA).

### Statistical analysis

All data were analyzed using Graph Pad Prism software (GraphPad Software, San Diego, CA, USA). The differences considered statistically significant were indicated as *P < 0.05, **P < 0.01, and ***P < 0.001. A two-tailed Student t test was used for pairwise comparisons.

## Electronic supplementary material

Supplemental information
