# Supplementary material for: Expression and role of VLA-1 in resident memory CD8 T cell responses to respiratory mucosal viral-vectored immunization against tuberculosis
Source: Sci Rep. 2017 Aug 25;7:9525. doi: 10.1038/s41598-017-09909-4 (PMC5573413; doi:10.1038/s41598-017-09909-4)
Supplement: Supplementary file 1 — Supplemental information [file 41598_2017_9909_MOESM1_ESM.pdf]

Expression and role of VLA-1 in resident memory CD8 T cell responses to respiratory mucosal viral-vectored immunization against tuberculosis

Siamak Haddadi[1,2], Niroshan Thanthrige-Don[1,2], Sam Afkhami[1,2,], Amandeep Khera[1,2,], Mangalakumari Jeyanathan[1,2,3], and Zhou Xing[1,2,3]*

[1]McMaster Immunology Research Centre, Department of Pathology & Molecular Medicine and [2]Michael G. DeGroote Institute for Infectious Disease Research, McMaster University, Hamilton, Ontario, Canada

[3]Joint senior authors

*Corresponding author:

Dr. Zhou Xing, Rm.4012-MDCL, Department of Pathology & Molecular Medicine,

McMaster University, 1280 Main Street West, Hamilton, ON L8S 4K1 Canada

E-mail: xingz@mcmaster.ca

Supplemental materials

Supplementary Table 1. Genes encoding for chemokine receptors, integrin heterodimers, and some activation makers implicated in T cell trafficking, maintenance and differentiation were profiled in FACS sorting-purified Ag-specific (Ag85A-tetramer-positive) CD8 T cells by custom-made PCR array.

**Chemokine receptors**	
**Genes**	**Proteins**
Ccr1	CCR1
Ccr2	CCR2
Ccr3	CCR3
Ccr4	CCR4
Ccr5	CCR5
Ccr6	CCR6
Ccr7	CCR7
Ccr8	CCR8
Ccr9	CCR9
Ccr10	CCR10
Il8ra	CXCR1
Il8rb	CXCR2
Cxcr3	CXCR3
Cxcr4	CXCR4
Cxcr5	CXCR5
Cxcr6	CXCR6
Cxcr7	CXCR7
Cx3cr1	CX3CR1
Xcr1	GPR5

**Effector molecules**	
**Genes**	**Proteins**
Sell	CD62L
Selplg	CD162
Cd44	CD44
Cd2	CD2/LFA-2
Cd5	CD5/Ly-1
Il2ra	CD25
Cd38	CD38
Il7r	CD127
Cd27	CD27
Cd69	CD69
Foxp3	FOXP3
Il2	IL-2
Prf1	Perforin
Gzma	GranzymeA
Gzmb	Granzyme B

**Integrin subunits**	
**Genes**	**Proteins**
itga1	CD49A (α1)
itga2	CD49B (α2)
Itga4	CD49D (α4)
itgae	CD103 (αE)
Itgal	CD11a (αL)
Itgb1	CD29 (β1)
Itgb2	CD18 (β2)
Itgb7	Ly69 (β7)

Supplementary Figure 1

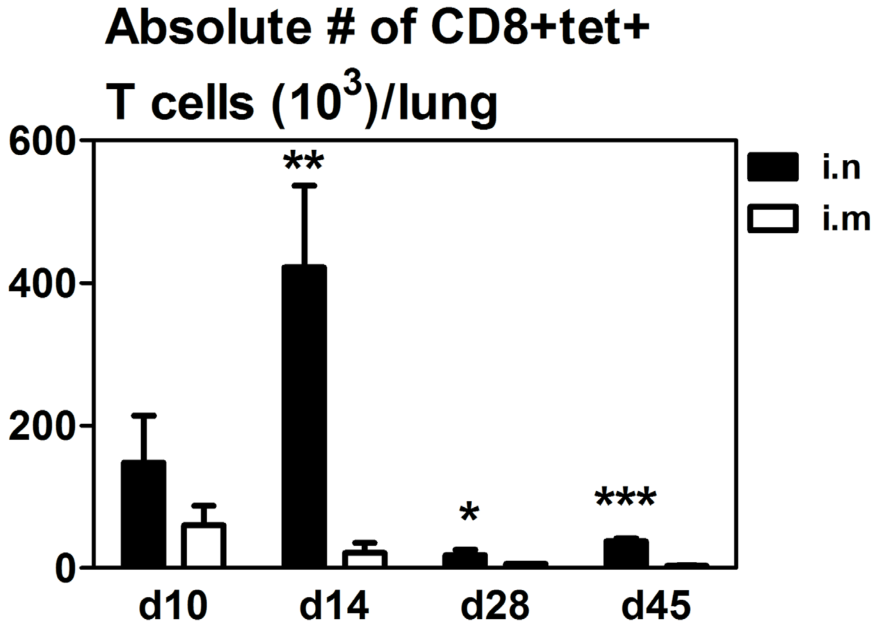

Kinetics of Ag-specific tetramer-positive (tet+) CD8 T cell responses in the lung following viral-vectored respiratory mucosal immunization. Mice were inoculated intranasally (i.n.) or intramuscularly (i.m.) with viral-vectored vaccine and lung mononuclear cells were isolated at designated time points (d10, d14, d28, or d45) post-immunization and immunostained to for Ag-specific CD8 T cells and analyzed using flow cytometry. Bar graph showing absolute numbers of Ag-specific CD8 T cells (CD8+tet+) at designated time points. Data are presented as the Mean ± S.E.M. of three mice per group per time point, representative of three independent experiments. *P<0.05, **P<0.01, ***P<0.001 compared with i.m. route of immunization.

Supplementary Figure 2

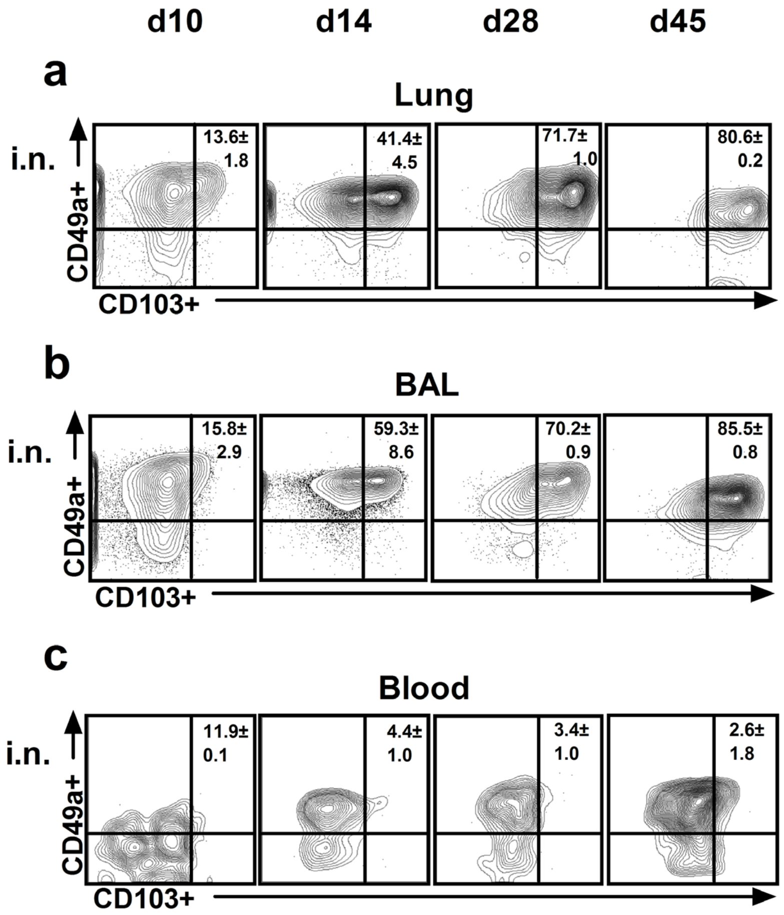

Kinetics of responses of Ag-specific CD8 T cells co-expressing both CD49a and CD103 following viral-vectored respiratory mucosal immunization. Mononuclear cells were isolated at designated time points (d10, d14, d28, or d45) from lung (a), BAL (b) and peripheral blood (c) post-i.n. immunization and immunostained to for tetramer, CD103 and CD49a and analyzed using flow cytometry. Dot plots showing frequencies of such T cells out of total Ag-specific CD8 T cells (tet+). Data are presented as the Mean ± S.E.M. of three mice per group per time point, representative of three independent experiments.

Supplementary Figure 3

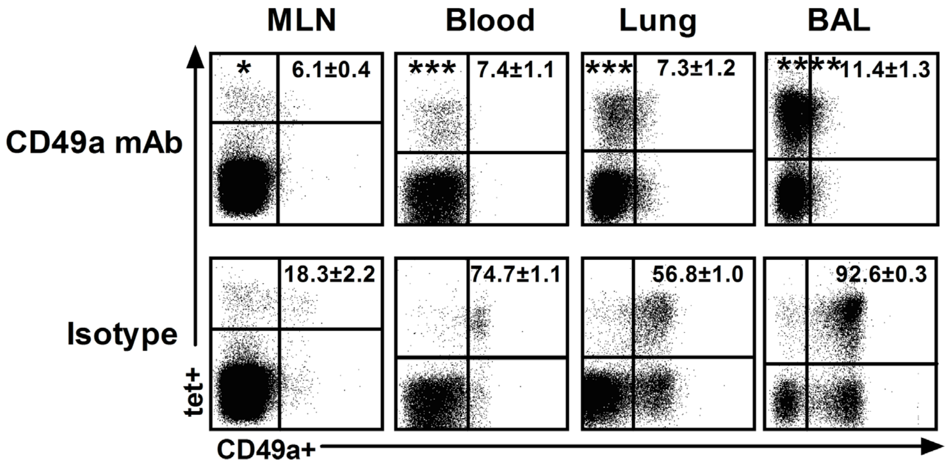

Efficiency of VLA-1 blockade following in vivo anti-CD49a blocking mAb treatment. Mononuclear cells were isolated from mediastinal lymph nodes (MLN), peripheral blood, lung, and BAL of mice that received viral-vectored respiratory mucosal immunization and treated with CD49a blocking mAb or isotype control antibody for 6 days. Cells were immunostained for tetramer and CD49a and analyzed using flow cytometry. Dot plots showing frequencies tet+CD49a+ CD8 T cells out of total Ag-specific CD8 T cells (tet+). Data are presented as the Mean ± S.E.M. of three mice per group from one experiment.

Supplementary Figure 4

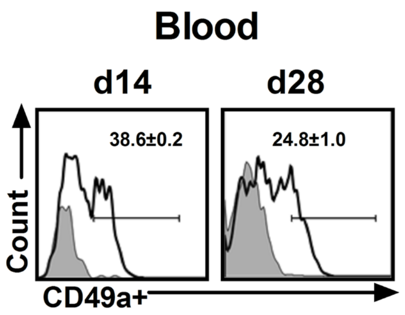

Expression of VLA-1 on replication-defective viral vector parenteral immunization-induced Ag-specific CD8 T cells in blood in different phases of T cell responses. Mononuclear cells were isolated at designated time points (d14 and d28) from peripheral blood post-i.m. immunization and immunostained for tetramer and CD49a and analyzed using flow cytometry. Dot plots showing frequencies of such T cells out of total Ag-specific CD8 T cells (tet+). Data are presented as the Mean ± S.E.M. of three mice per group per time point, representative of three independent experiments.
